# Differentiation of Cervical Spine Osteoradionecrosis and Bone Metastasis After Radiotherapy Detected by Bone Scan in Patients With Nasopharyngeal Carcinoma: Role of Magnetic Resonance Imaging

**DOI:** 10.3389/fonc.2020.00015

**Published:** 2020-01-24

**Authors:** Xi Zhong, Li Li, Bingui Lu, Hainan Zhang, Lu Huang, Xinjia Lin, Jiansheng Li, Jian Zhang

**Affiliations:** ^1^Department of Radiology, Affiliated Cancer Hospital & Institute of Guangzhou Medical University, Guangzhou, China; ^2^Department of Otolaryngology, The Third Affiliated Hospital of Guangzhou Medical University, Guangzhou, China; ^3^Department of Radiation Oncology, Affiliated Cancer Hospital & Institute of Guangzhou Medical University, Guangzhou, China

**Keywords:** nasopharyngeal carcinoma, osteoradionecrosis, cervical spine, magnetic resonance imaging, bone scan

## Abstract

**Background:** Osteoradionecrosis (ORN) of the cervical spine is a serious complication after radiotherapy (RT), which may show increased radiotracer uptake on a bone scan (BS) and be mistaken as metastasis. We aimed to assess the value of magnetic resonance imaging (MRI) in the differentiation of cervical spine ORN from bone metastasis after RT detected by BS in nasopharyngeal carcinoma (NPC).

**Methods:** In this retrospective study, 35 NPC patients who had undergone RT were enrolled, of whom 21 patients showed cervical spine ORN and 14 showed bone metastasis. New areas of increased radiotracer uptake in the cervical spine on a BS were noted in all patients, following which the patients underwent neck MRI for further assessment. Two radiologists independently reviewed two sets of images including a BS set and an MRI set (MRI with BS) and reached a consensus. The diagnostic sensitivity, specificity, and accuracy for ORN detection were calculated, and interobserver agreement was evaluated using the kappa test.

**Results:** A total of 75 cervical spine lesions were identified (44, ORN; 31 metastases). The BS set analysis showed that the diagnostic sensitivity, specificity, and accuracy were only 38.6, 48.3, and 42.7%, respectively, for differentiation of cervical spine ORN from bone metastasis. On the other hand, the MRI set analysis showed that the diagnostic sensitivity, specificity, and accuracy increased to 86.4, 90.3, and 88.0%, respectively. The interobserver agreement for the MRI set was determined to be very good (κ = 0.92).

**Conclusion:** MRI is a reliable technique for the further discrimination of emerging cervical spine lesions after RT detected by BS. Furthermore, it could be a better differential diagnosis technique for distinguishing ORN from metastasis and may help avoid a wrong assignment of the patient to a metastatic stage with indication for treatment with supplemental toxicity and a subsequent palliative strategy.

## Introduction

Nasopharyngeal carcinoma (NPC) is a malignant tumor with a very unique geographic distribution; it is mainly prevalent in southern China and Southeast Asia (1, 2). In the regions with NPC prevalence, NPC has been reported in approximately 50 per 100,000 persons annually and this incidence is almost 50 times higher than that in western countries (2). Radiotherapy (RT) has been identified as the primary therapeutic method for NPC, and post-RT adverse events have drawn a great deal of attention.

Post-RT adverse events in patients with NPC involving the central nervous system, including radiation encephalopathy, diffuse white matter injury, and optic neuritis, have been well-described (3). Osteoradionecrosis (ORN), with an incidence of 10.1%, is also a common complication after RT; it frequently develops in the mandible, maxilla, and skull base (4–6). Nevertheless, ORN in the cervical spine has been regarded as a rare complication, although the cervical vertebrae are often included in the irradiation field (6–9). Sometimes, cervical spine ORN may be misdiagnosed as bone metastasis, which may lead to patients accepting an unnecessary biopsy or harmful chemoradiotherapy (10–12).

Modern medical imaging has played a great role in the detection and characterization of bone disorders. Bone scan (BS) is considered as a sensitive enough technique for the detection of bone marrow diseases and has been widely applied for monitoring bone metastasis in patients with a malignancy (13). However, BS has a limitation in terms of specificity, because RT-induced bone complications, acute osteoporotic fractures, and the metastases may all result in increased uptake (14–16). MRI is quite sensitive for the detection of reactive marrow edema and fatty changes, and very helpful for the detection of soft-tissue masses. MRI has been proven to be valuable for distinguishing benign and malignant compression spinal fractures (17, 18), and differentiating RT-induced insufficiency fractures from metastases (14). However, few studies have assessed the MRI findings of ORN (10–12), and the role of MRI in the distinction of cervical spine ORN and metastasis remains unknown. Thus, the purpose of this study was to assess the additional value of MRI in the identification of cervical spine ORN and metastasis after radiotherapy in patients with NPC detected by BS.

## Materials and Methods

### Patient Samples

**Figure 1:**
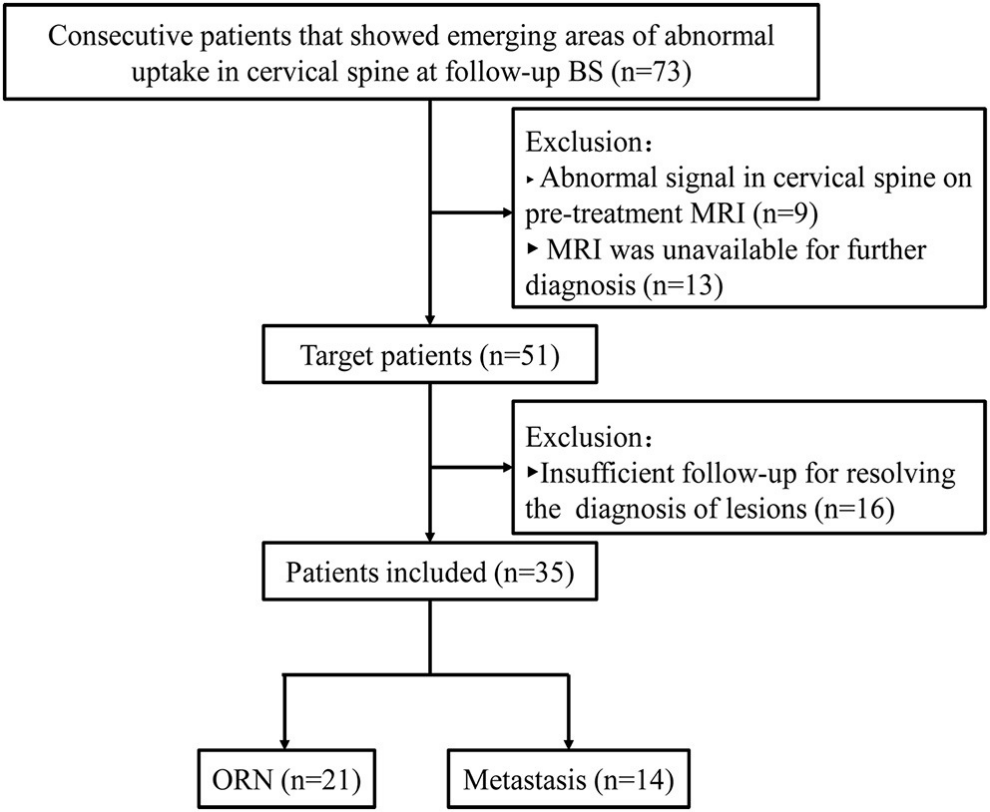
Flowchart of the study population.

The ethics committee of Affiliated Cancer Hospital & Institute of Guangzhou Medical University approved this retrospective study, and the requirement of patients' informed consent was waived. Between January 2013 and December 2016, data were reviewed for 2046 consecutive patients with pathology-proven NPC after RT. We found 73 patients showed new cervical spine lesions at follow-up BS. The following patients were included in this study: (1) patients with emerging areas of abnormal uptake in the cervical spine; (2) patients who underwent neck MRI for further assessment; and (3) patients for whom sufficient clinical and MRI follow-up data were available to resolve the diagnosis of lesions. The following patients were excluded from the study: (1) patients with an abnormal signal in the cervical spine on pretreatment MRI, (2) patients for whom MRI data were unavailable after lesion detection by BS; and (3) patients in whom the diagnosis of lesions could not be confirmed. Finally, 35 NPC patients who had undergone RT were enrolled, of whom 21 patients showed cervical spine ORN and 14 showed bone metastasis. The patient selection flowchart is shown in Figure 1.

### MRI Acquisition

MRI was performed using a 1.5-T system unit (Achieva, Philips) with a 16-channel head-neck combined coil. The sequences included axial turbo spin-echo (TSE) T1-weighted imaging (T1WI); the parameters were as follows: TR/TE, 545/14 ms; field of view (FOV), 23 cm; slice thickness, 4 mm; gap, 4 mm; and matrix size, 328 × 220. Axial TSE T2-weighted imaging (T2WI) was also performed, the parameters for which were as follows: TR/TE, 3193/80 ms; FOV, 23 cm; slice thickness, 5 mm; gap, 5 mm; and matrix size, 228 × 185. Furthermore, coronal fat-suppression (FS) T2-weighted imaging (FS-T2WI) was performed, the parameters for which were as follows: TR/TE, 3224/165 ms; FOV, 26 cm; slice thickness, 5 mm; gap, 5 mm; and matrix size, 312 × 163. Axial and sagittal FS contrast-enhanced T1WI was also employed; parameters of contrast-enhanced T1WI were the same as those for the pre-enhanced T1WI in addition to the FS technique. Enhanced T1WI was performed after the patients had received intravenous gadopentetate dimeglumine (Magnevist, Bayer Schering) at a dose of 0.1 mmol/kg.

### BS Acquisition

Whole-body BS was performed using a Philips SPECT scanner (Netherlands). Both anterior and posterior whole-body bone images were obtained 3 h after intravenous injection of 15–25 mCi 99 mTc-MDP; the scan parameters were as follows: matrix, 256 × 1,024; scanning speed, 15 cm/min; and acquisition energy window, 140 keV (±7.5%).

### Image Analysis

All images were independently reviewed by two radiologists (observer 1, J.S.L, with 12 years of experience; observer 2, B.G.L, with 15 years of experience) and an agreement was reached. Two sets of images were designed to differentiate cervical spine ORN and metastasis, including a BS (BS images alone) set and an MRI set (MR images and BS images). Observers were only aware of the RT history but were blinded to patients' other clinical records, other imaging examination (CT, PET/CT) data, and the final diagnosis. Regarding the qualitative diagnosis, all cervical spine lesions were classified on a three-point scale as benign, malignant, or equivocal.

In the BS analysis, the observers were requested to record the abnormal uptake location and number of the cervical spine, as well as whether abnormal uptake was noted in other anatomical locations except for the cervical spine. If focal radiotracer uptake was greater than that in the anterior iliac spine, the lesion was classified as malignant (13, 17). A lesion was classified as benign if radiotracer uptake was equal to or lower than that in the anterior iliac spine and no abnormal uptake was noted in other anatomical locations except for the cervical spine. If a lesion was not classified as malignant or benign, it was classified as equivocal.

In the MRI set analysis, the number, location, MR signal characteristics (bone marrow edema change, degree, and pattern of enhancement), vertebral destruction, and body collapse of cervical spine lesions were recorded; any associated paravertebral soft-tissue masses were also recorded. In addition, other ancillary features were also documented, including abnormal enhancement or necrosis of the paravertebral muscle, abnormal neck lymphadenopathy (short axis larger than 1 cm), and radiation encephalopathy (REP). Regarding the qualitative diagnosis, the criteria for lesion classification as malignant were as follows: (1) presence of a distinct soft-tissue mass with abnormal enhancement on MRI (10, 17); and (2) absence of a distinct soft-tissue mass but presence of marked vertebral and heterogeneous enhancement on MRI and involvement of abnormal neck lymphadenopathy or meeting of the BS criteria for malignancy. The criteria for lesion classification as benign were as follows: (1) presence of only reactive marrow edema change with or without paravertebral muscle edema (19, 20); and (2) presence of an abnormal signal change on unenhanced images and homogeneous enhancement on contrast-enhanced images, and meeting of the BS criteria for benign lesions. If a lesion was not classified as malignant or benign, it was classified as equivocal.

### Reference Standard

The final diagnosis of a cervical spine lesion was based on all available imaging investigations, clinical data, and follow-up MRI data for at least 6 months. The reference standard was as follows (10, 19): (1) lesions that shrank or remained stationary at MRI for more than 6 months without radiotherapy and/or chemotherapy were interpreted as ORN; and (2) lesions with progressive enlargement that presented as soft-tissue masses or distinctly regressed after radiotherapy and/or chemotherapy were identified as metastases. If a lesion's nature could not be confirmed by a follow-up procedure, it was excluded.

### Statistical Analysis

Categorical data were expressed as numbers and frequencies (%), and continuous data were expressed as median and range. Interobserver agreement between observers was assessed using the kappa test and defined as follows (21): 1 ≥ k > 0.8, very good; 0.8 ≥ k > 0.6, good; 0.6 ≥ k > 0.4, moderate; 0.4 ≥ k > 0.2, fair; and 0.2 ≥ k > 0, poor. The diagnostic sensitivity, specificity, and accuracy were calculated based on the diagnostic scale. Pearson chi-square test (or Fisher test) was used for comparing differences in the incidence of imaging features between ORN and bone metastasis. Statistical tests were performed using SPSS 16.0 (SPSS Inc., Chicago, IL, USA); *P* < 0.05 indicated statistical significance.

## Result

### Patient Characteristics

A total of 35 patients were included, 60% (21/35) of whom were diagnosed with cervical spine ORN and 40% (14/35) were identified as metastasis. Among the patients with ORN, 23.8% (5/21) had undergone repeat RT to the neck, 66.7% (14/21) had developed ORN at multiple locations, and 76.2% (16/21) were symptomatic. In the patients with metastasis, 7.1% (1/14) had undergone repeat RT to the neck, 57.1% (8/14) had multiple lesions, and 57.1% (8/14) were symptomatic. Detailed patient characteristics are shown in Table 1.

**Table 1:** Characteristics of NPC patients after RT enrolled in the study.

**Characteristics**	**ORN (*n* = 21)**	**Metastasis (*n* = 14)**
Median age (range)	51 (30–80) years	43 (23–65) years
Male/Female	17/4	9/5
RT dose (rang)	81 (68–154) Gy	74 (58–128) Gy
Median interval between RT and lesion detection (rang)	10 (2–96) months	10 (4–24) months
**Overall stage**^**#**^		
I	6 (28.6%)	1 (7.2%)
II	8 (38.1%)	3 (21.4%)
III	5 (23.8%)	7 (50.0%)
IV	2 (9.5%)	3 (21.4%)
**Received repeat rt to the neck**		
Yes	5 (23.8%)	1 (7.2%)
No	16 (76.2%)	13 (92.8%)
Clinical symptoms^*^		
Neck pain	16 (76.2%)	9 (64.3%)
Infection	6 (28.6%)	1 (7.2%)
Asymptomatic	5 (23.8%)	6 (42.8%)
**Involvement of multiple lesions**		
Yes	14 (66.7%)	8 (57.1%)
No	7 (33.3%)	6 (42.9%)

# *According to the 7th UICC/AJCC staging system*.

* *A patient may show neck pain with infection*.

*ORN, osteoradionecrosis*.

### Numbers and Locations of ORN and Metastasis of the Cervical Spine

**Figure 2:**
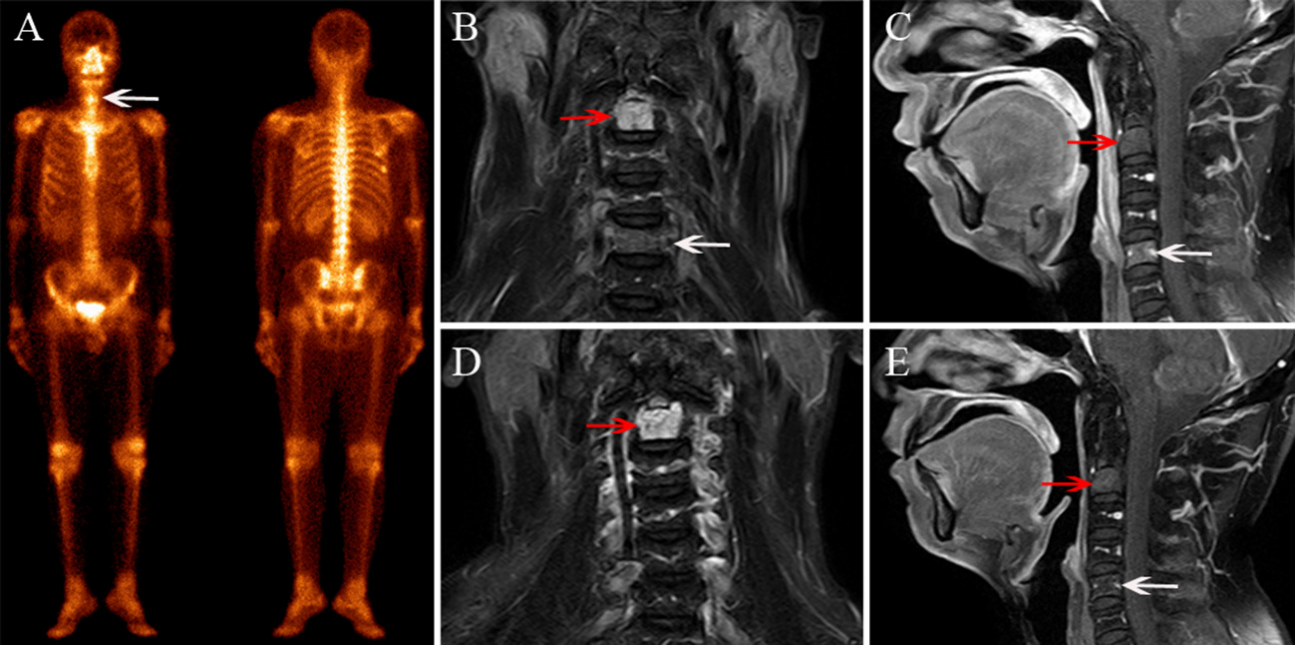
A 42-year–old male diagnosed with cervical spine ORN after RT for NPC. **(A)** BS shows increased radiotracer uptake in C5 (white arrow) and the seventh right rib. **(B)** Coronal FS T2–weighted image shows hyperintensity in the C2 (white arrow) and C5 vertebra (red arrow). **(C)** Sagittal contrast-enhanced T1-weighted image shows mild enhancement in C2 and moderate enhancement in C5. **(D,E)** MRI follow-up examination after 8 months, coronal FS T2-weighted image **(D)**, and sagittal contrast-enhanced T1-weighted image **(E)** show that the signal change in C2 has remained stationary and the abnormal signal in C5 has disappeared.

**Figure 3:**
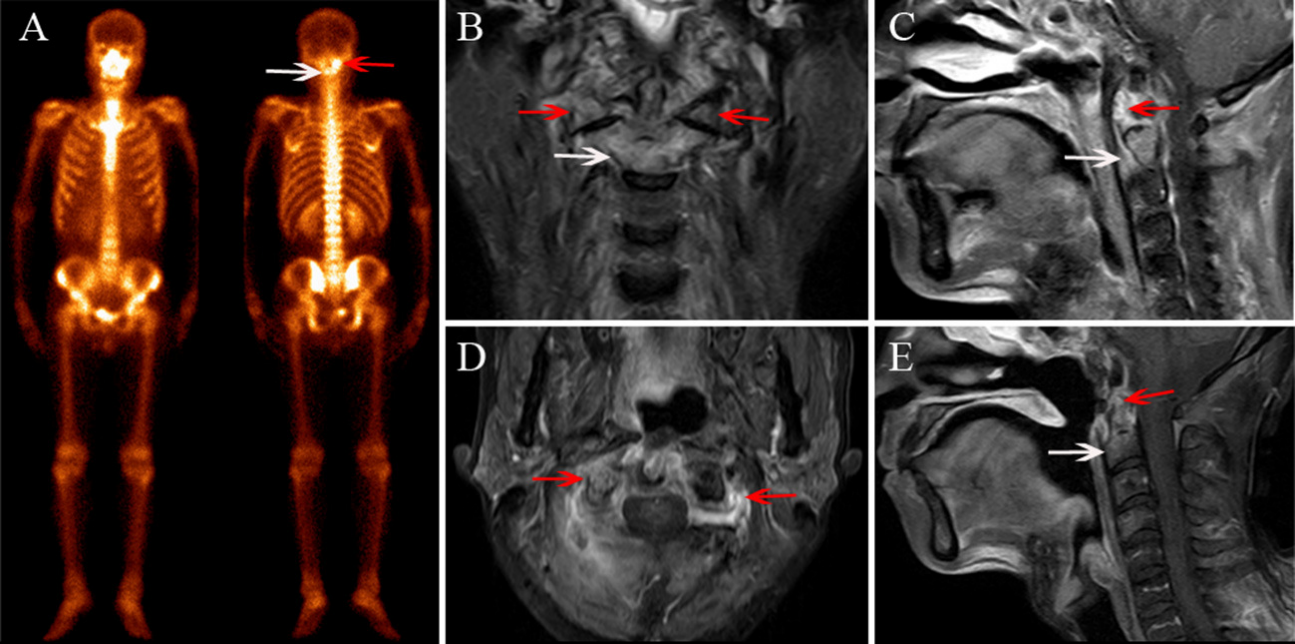
A 56-year-old female diagnosed with cervical spine ORN after RT for NPC. **(A)** BS shows increased radiotracer uptake in C1 (red arrow) and C2 (white arrow). **(B)** Coronal FS T2-weighted image shows hyperintensity in C2 (white arrow) and the bilateral aspect of C1 (red arrow). **(C)** Sagittal contrast-enhanced T1-weighted image shows marked enhancement in lesions. **(D)** Sagittal contrast-enhanced T1-weighted image shows paravertebral muscle edema and marked enhancement change. **(E)** MRI follow-up after 6 months and a sagittal contrast-enhanced T1-weighted image shows that the lesion area has shrunken and the enhancement has declined.

**Figure 4:**
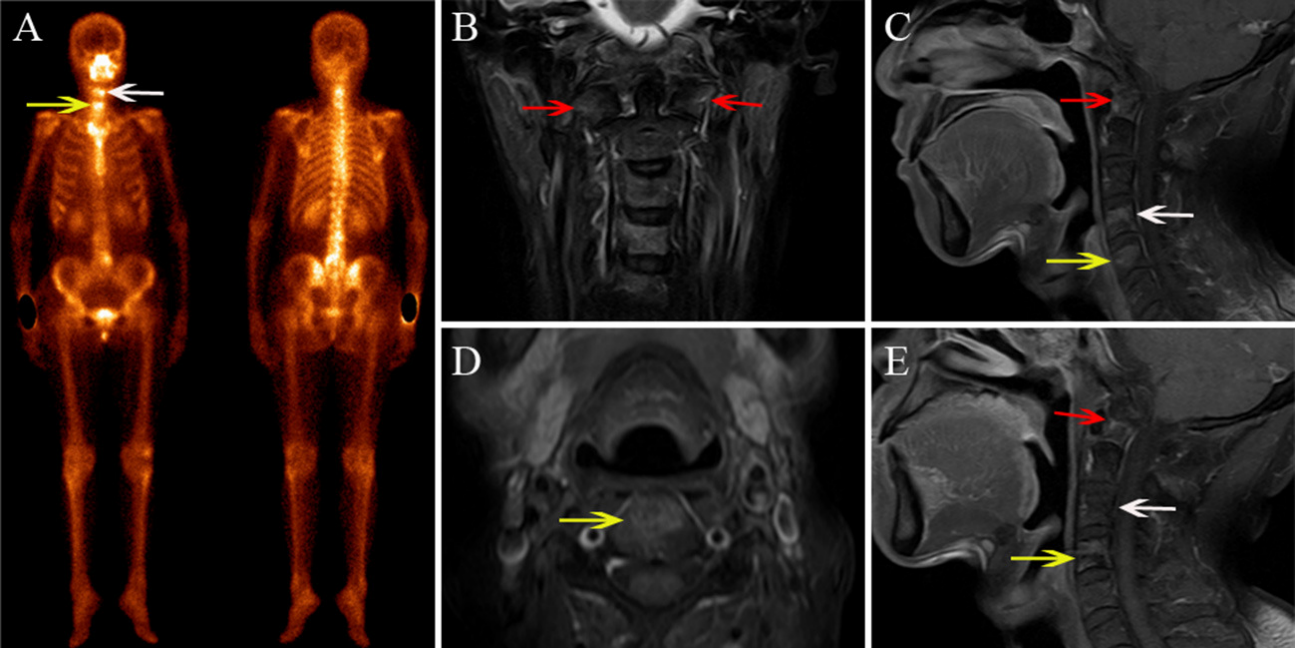
A 48-year-old male diagnosed with cervical spine ORN after RT for NPC. **(A)** BS shows increased radiotracer uptake in C4 (white arrow) and C5 (yellow arrow). **(B)** Coronal FS T2-weighted image shows hyperintensity in the bilateral aspect of C1 (red arrow), C4, and C5. **(C,D)** Sagittal enhanced T1-weighted image **(C)** and axial enhanced T1-weighted image **(D)** show mild enhancement in the lesions. **(E)** MRI follow-up after 7 months; the sagittal contrast-enhanced T1-weighted image shows that the area of the lesions has shrunken and the enhancement has declined in C4 and C5, while the signal change of C1 has remained stationary.

Based on the reference standard, a total of 75 cervical spine lesions were identified, including 44 cases of ORN and 31 of metastases. As shown in Table 2, ORN most frequently developed in C1/C2 (Figures 2–4), accounting for 47.7% (21/44) of all lesions; the detailed locations of the ORN were as follows: C1, 10 sites; C2, 11 sites; C3, 5 sites; C4, 6 sites; C5, 6 sites; C6, 5 sites; and C7, 1 site. Cervical spine metastases were located at C1 (7 sites), C2 (5 sites), C3 (5 sites), C4 (3 sites), C5 (5 sites), C6 (4 sites), and C7 (2 sites).

**Table 2:** Number and locations of the ORN and metastasis of the cervical spine.

**Location**	**ORN**	**Metastasis**	**Total**
C1	10	7	17
C2	11	5	16
C3	5	5	10
C4	6	3	9
C5	6	5	11
C6	5	4	9
C7	1	2	3
Total	44	31	75

*ORN, Osteoradionecrosis*.

### BS Findings and Diagnostic Performance

**Figure 5:**
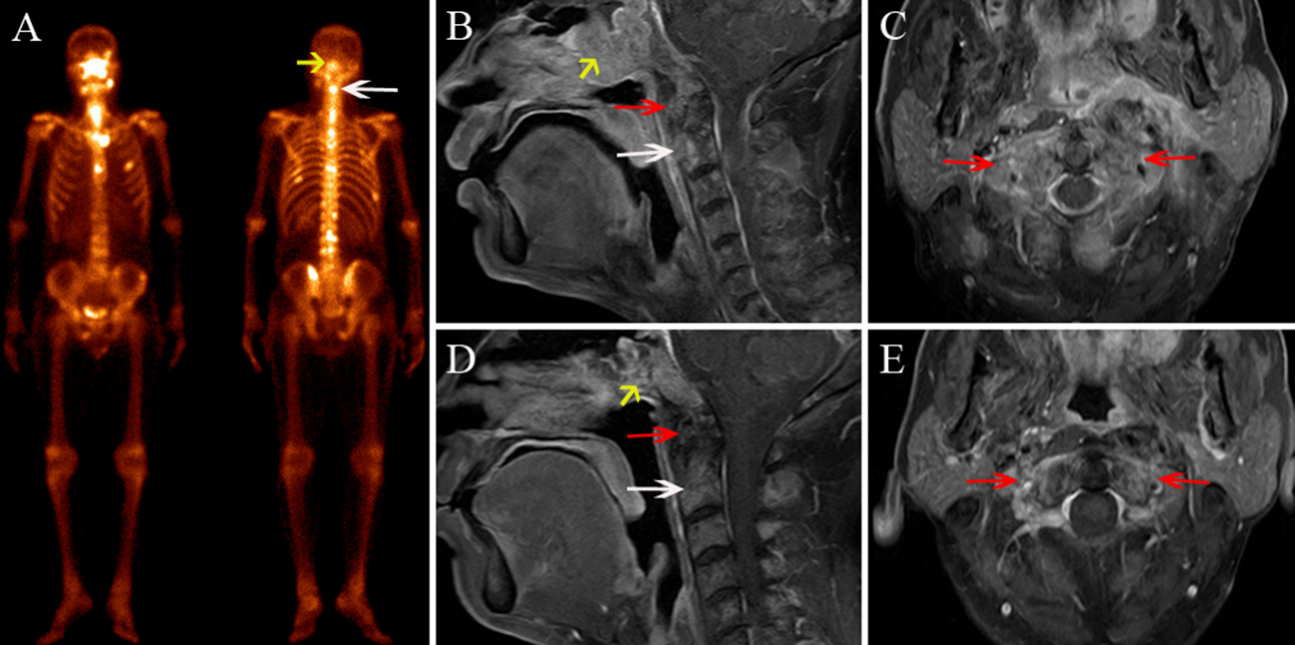
A 53-year-old male diagnosed with skull base recurrence combined with cervical spine metastasis after RT for NPC. **(A)** BS shows increased radiotracer uptake in the skull base (yellow arrow) and C2 (white arrow), multiple ribs, thoracic vertebrae, and lumbar vertebrae, also show increased radiotracer uptake. **(B)** Sagittal contrast-enhanced T1-weighted imaging shows an enhanced soft-tissue mass in the skull base (yellow arrow), C1 (red arrow), and C2 (white arrow). **(C)** Axial contrast-enhanced T1-weighted image shows enhanced soft-tissue mass and bone destruction in the bilateral aspect of C1 (red arrow). **(D,E)** In an MRI follow-up 3 months after RT, the sagittal enhanced T1-weighted image **(D)** and axial enhanced T1-weighted image **(E)** show that the lesion area has shrunken and the enhancement has declined.

**Figure 6:**
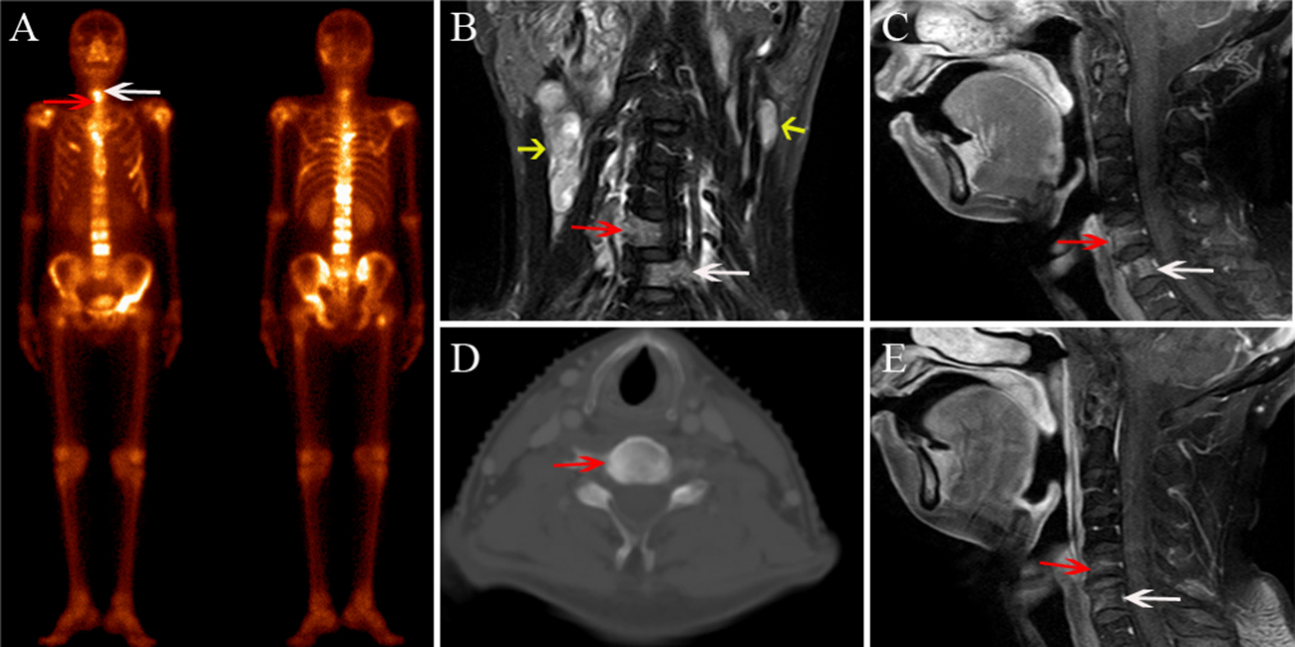
A 68-year-old female diagnosed with bone metastasis after radiotherapy for NPC. **(A)** BS shows increased radiotracer uptake in C5 (white arrow) and C6 (red arrow); multiple ribs, thoracic vertebrae, lumbar vertebrae, and the right femoral neck also show increased radiotracer uptake. **(B)** Coronal FS T2-weighted image shows hyperintensity in C5 (white arrow) and C6 (red arrow), and multiple enlarged lymph nodes in both sides of the neck (yellow arrow). **(C)** Sagittal contrast-enhanced T1-weighted image shows marked and heterogeneous enhancement in C5 and C6. **(D)** CT localization image shows an osteogenic change in C5. **(E)** In the MRI follow-up 2 months after RT, the sagittal contrast-enhanced T1-weighted image shows that the area of the lesions has shrunken and the enhancement has declined.

As shown in Table 3, in the patient-wise analysis, the incidence of involvement with abnormal uptake in other anatomical locations except for the cervical spine in the metastasis group (Figures 5, 6) was significantly higher than that in the ORN group (Figure 2) [64.3% (9/14) vs. 23.8% (5/21)], *P* = 0.016). In the lesion-wise analysis, only 61.4% (27/44) of the ORNs and 80.6% (25/31) of the metastases were detected on BS. The incidence of focal radiotracer uptake greater than the anterior iliac spine in metastases (Figures 5, 6) was significantly higher than that in ORNs (Figure 3) [48.4% (15/31) vs. 11.4% (5/44), *P* < 0.001].

**Table 3:** Findings of cervical spine lesions on BS and MRI.

**Imaging findings**	**ORN**	**Metastasis**	***P***
**BS findings**			
Patient-wise analysis (*n*)	21	14	
**Abnormal uptake at other bone sites**			
Yes	5 (23.8%)	9 (64.3%)	0.016
No	16 (72.2%)	5 (35.7%)	
Lesion-wise analysis (*n*)	44	31	
Detection rate	27 (61.4%)	25 (80.6%)	0.07
Radiotracer uptake greater than that in the anterior iliac spine	5 (11.4%)	15 (48.4%)	< 0.001
**MRI findings**			
Patient-wise analysis (*n*)	21	14	
**Cervical lymphadenopathy**			
Yes	2 (9.5%)	11 (78.5%)	< 0.001
No	19 (90.5%)	6 (42.9%)	
**Radiation encephalopathy**			
Yes	2 (9.5%)	0 (0%)	0.145
No	19 (90.5%)	14 (100%)	
Lesion-wise analysis (*n*)	44	31	
Vertebral marrow edema	42 (95.5%)	23 (74.2%)	0.007
Vertebral enhancement	42 (95.5%)	31 (100%)	0.141
Vertebral destruction	6 (13.6%)	10 (32.3%)	0.054
Vertebral soft-tissue mass	4 (9.1%)	12 (38.7%)	0.002
Vertebral body collapse	1(2.3%)	3 (9.7%)	0.163
Paravertebral muscle edema	20 (45.5%)	6 (19.4%)	0.017

*ORN, Osteoradionecrosis*.

*Numbers in parentheses were used to calculate percentages; we calculated P-values by using the Pearson chi-square test (or Fisher test); P < 0.05 indicates a significant difference*.

The qualitative assessment showed that 17 lesions of the 27 ORNs detected on BS were interpreted as benign, 5 lesions were classified as malignant, and the remaining 5 lesions showed equivocal findings; 15 lesions of the 25 metastases detected on BS were interpreted as malignant, 2 lesions were classified as benign, and the remaining 8 lesions showed equivocal findings. The diagnostic sensitivity, specificity and accuracy in the discrimination of ORN from metastasis were 38.6% (17/44), 48.3% (15/31), and 42.7% (32/75), respectively. The interobserver agreement between the two observers in the BS set was determined to be good (*k* = 0.77).

### MRI Findings and Additional Diagnostic Performance

As shown in Table 3, in the patient-wise analysis, the incidence of involvement with cervical lymphadenopathy in the metastasis group (Figure 6B) was significantly higher than that in the ORN group [78.5% (11/14) vs. 9.5% (2/21), *P* = 0.002]. In the lesion-wise analysis, all lesions were detected by MRI, and the incidences of vertebral marrow edema (Figures 2B, 4B) and paravertebral muscle edema (Figure 3D) in ORNs were significantly higher than those in metastases (95.5% (42/44) vs. 74.2% (23/31), *P* = 0.007; 45.5% (20/44) vs. 19.4% (6/31), *P* = 0.017, respectively]. In contrast, the incidence of vertebral soft-tissue mass in metastases (Figures 5B,C) was significantly higher than that in ORNs [38.7% (12/31) vs. 9.1% (4/44), *P* = 0.002].

For qualitative assessment, in the 44 cases showing ORNs, 38 lesions were interpreted as benign, 3 lesions were classified as malignant, and the remaining 5 lesions were considered to show equivocal findings. Of the 31 metastases, 28 lesions were interpreted as malignant, 3 lesions were classified as equivocal, and no lesion was classified as benign. For the discrimination of ORN from metastasis, with the addition of MRI, the diagnostic sensitivity, specificity, and accuracy increased to 86.4% (38/44), 90.3% (28/31), and 88.0% (66/75), respectively. The interobserver agreement in the MRI set was determined to be very good (*k* = 0.92).

## Discussion

Accurately distinguishing cervical spine ORN from metastasis is crucial in clinical practice. This study demonstrated that both ORN and metastasis could show increased radiotracer uptake on a BS, and BS showed comparatively low detection sensitivity and classification efficiency in the discrimination of ORN and metastasis. MRI showed additional value when used along with BS; the combined approach showed a better lesion detection rate and improved diagnostic sensitivity and specificity.

BS has been considered as one of the most common and accessible modern imaging procedures for monitoring bone metastasis in patients with malignancy. BS shows enough sensitivity for detecting metastasis, but it shows several limitations in specificity because many benign bone tumors, infections, and degenerative disease may also show abnormal radiotracer uptake (13, 15, 16, 22). Recently, BS has been applied for detecting RT-induced bone complications and has shown variable sensitivity in the detection of insufficiency fractures after RT for pelvic malignancy, with a detection rate ranging from 40 to 87.5% (14, 15, 23). In this study, BS showed a detection sensitivity of 61.4% for detecting ORN. Thus, novel imaging techniques need to be applied to improve the detection sensitivity of ORN.

MRI is an important alternative imaging modality for further assessment of vertebral benign and malignant diseases. However, the value of MRI in the characterization of cervical spine ORN has not been well-described. To date, only few studies have discussed the MRI features of ORN that occurred in the upper (C1/C2) cervical spine (10–12), and ORN in other anatomical locations of the cervical spine was also only reported in some case reports (24–26). In this study, we found that ORN more frequently developed in C1/C2, which may be attributable to the fact that C1/C2 is adjacent to the nasopharynx and may be more susceptible to RT. Our study also demonstrated that 66.7% of patients with ORN showed devolution with multiple vertebras, which was consistent with the findings of previous studies (10, 11).

We found some MRI findings that could facilitate the differential diagnosis of cervical spine ORN from metastasis. First, patients with metastasis tended to show the involvement of cervical lymphadenopathy, with almost 80% of the patients showing cervical spine metastasis combined with cervical lymphadenectasis, which was also consistent with the results obtained by Wu et al. (10). Second, reactive vertebral marrow edema was the most common feature for ORN, with up to 95.5% of ORNs showing vertebral marrow edema change, and the bone marrow edema in ORN was frequently asystematic and homogeneous, but the marrow edema in metastasis was more likely to be circumscribed and asymmetrical. Third, reactive paravertebral muscle edema was also a distinctive ancillary feature that may be associated with ORN. In addition, a vertebral soft-tissue mass was a reliable symptom for detecting metastasis. Although, King et al. (11) indicated that some cervical ORN patients may also have soft-tissue masses adjacent to the cervical spine, we found that these lesions were usually less bulky, and the signal intensity of edema tended to be extensive and rarely showed an occupied effect. These findings exhibited by ORN likely reflect localized infectious and inflammatory processes (19, 20, 27). Meanwhile, the soft-tissue involvement in ORN may be more symmetric than that in metastasis. In summary, accounting for these meaningful findings, MRI may be a reliable technique for distinguishing ORN from bone metastasis, and the possibility of aggressive radiotherapy for patients due to misdiagnosis may be effectively avoided with the involvement of MRI.

With respect to the diagnostic efficiency in the identification of cervical spine ORN and metastasis, BS showed limited value for differential diagnosis. In our study, the diagnostic sensitivity, specificity, and accuracy using BS alone were only 38.6, 48.3, and 42.7%, respectively. This may be attributed to the relatively low lesion detection sensitivity on BS and the fact that a large number of lesions detected were classified as equivocal. We found that the diagnostic sensitivity increased to 86.4% and specificity increased to 90.3% with the addition of MRI. Meanwhile, 38.6% of ORNs and 19.4% of metastases that were not detected on BS were found on MRI, and the frequency of equivocal lesions was also reduced. These results were similar to those of several previous studies that assessed the added value of MRI or SPECT/CT to BS alone in the discrimination of RT-induced pelvic insufficiency fracture and pelvic metastasis in cervical cancer patients (13–15, 22). Therefore, the results of our study indicated that MRI could be a noninvasive technique for further discrimination of new cervical spine lesions detected by BS.

This study had several limitations. First, puncture pathologic evaluation was not available due to the high probability of fracture or hemorrhage; thus we used standardized clinical and MRI follow-up to confirm ORN or metastasis. Second, this was a retrospective study with a limited sample performed in a single center. Further prospective studies with larger sample sizes are needed to validate the results. Third, only routine MRIs were used to assess the diagnostic efficacy, and the potential performance of some functional MR imaging techniques, such as diffusion-weighted imaging (DWI), and perfusion-weighted imaging (PWI), need to be Friday, January 10, 2020 1:47 pm explored further.

In conclusion, both cervical spine ORN and metastasis can show increased radiotracer uptake on a BS in patients with NPC after RT, and MRI showed additional value in identifying these cervical spine lesions. MRI could be a better differential diagnosis technique for distinguishing ORN from metastasis and may avoid wrong assignment of patients to a metastatic stage with indications for treatment modalities with supplemental toxicity and a subsequent palliative strategy.

## Data Availability Statement

The datasets generated for this study are available on request to the corresponding author.

## Ethics Statement

The studies involving human participants were reviewed and approved by Affiliated Cancer Hospital & Institute of Guangzhou Medical University. Written informed consent for participation was not required for this study in accordance with the national legislation and the institutional requirements. Written informed consent was not obtained from the individual(s) for the publication of any potentially identifiable images or data included in this article.

## Author Contributions

XZ and JZ: conceptualization, writing—original draft preparation, and supervision. LL, BL, and HZ: methodology. BL and JL: formal analysis. HZ, LH, and JL: investigation. XZ and LL: resources. LH and XL: data curation. JL and JZ: writing—review and editing. All authors read and approved the final manuscript.

### Conflict of Interest

The authors declare that the research was conducted in the absence of any commercial or financial relationships that could be construed as a potential conflict of interest.
